# Supplementary material for: EGFR Pathway Expression Persists in Recurrent Glioblastoma Independent of Amplification Status
Source: Cancers (Basel). 2023 Jan 21;15(3):670. doi: 10.3390/cancers15030670 (PMC9913094; doi:10.3390/cancers15030670)
Supplement: Supplementary file 1 [file cancers-15-00670-s001.zip › CRC-22-0269_Supplementary_Data.pdf]

Supplementary Information

Supplementary Figure S1. Scatterplot depicting correlation (Spearman's rho = 0.79, $p < 10^{-15}$) between gene expression across primary and recurrent tumours, across all genes and all samples. Due to the high number of genes across all samples, scatterplot is overlaid with heatmap indicating point density.

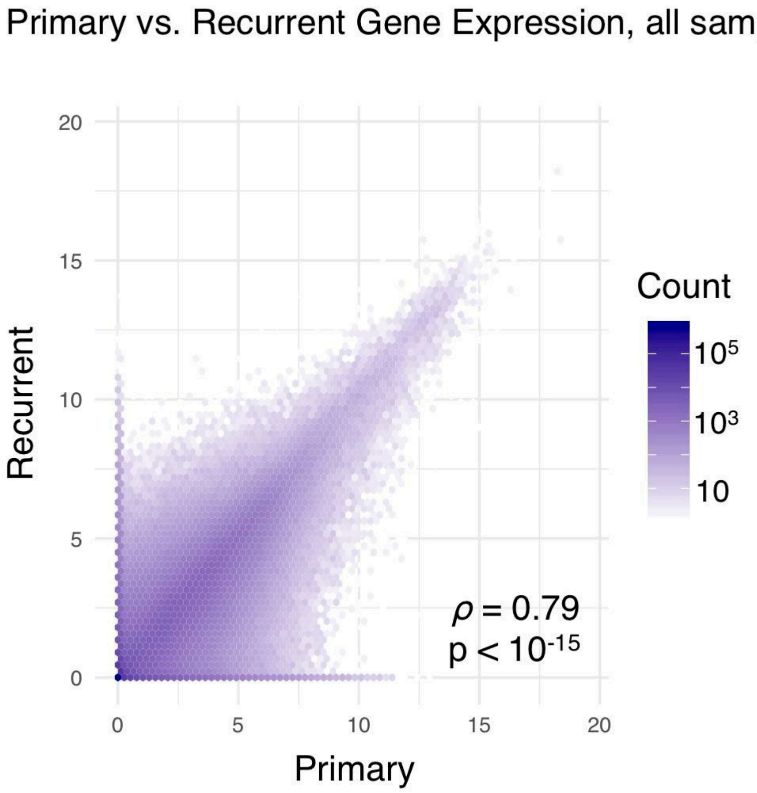

Supplementary Figure S2. Correction of batch effects. PCA plot describing post-filtering of relatively poorly expressed transcripts across all samples in primary and recurrent tumours, pre-batch correction **(A)**, and post-batch correction **(B)**.

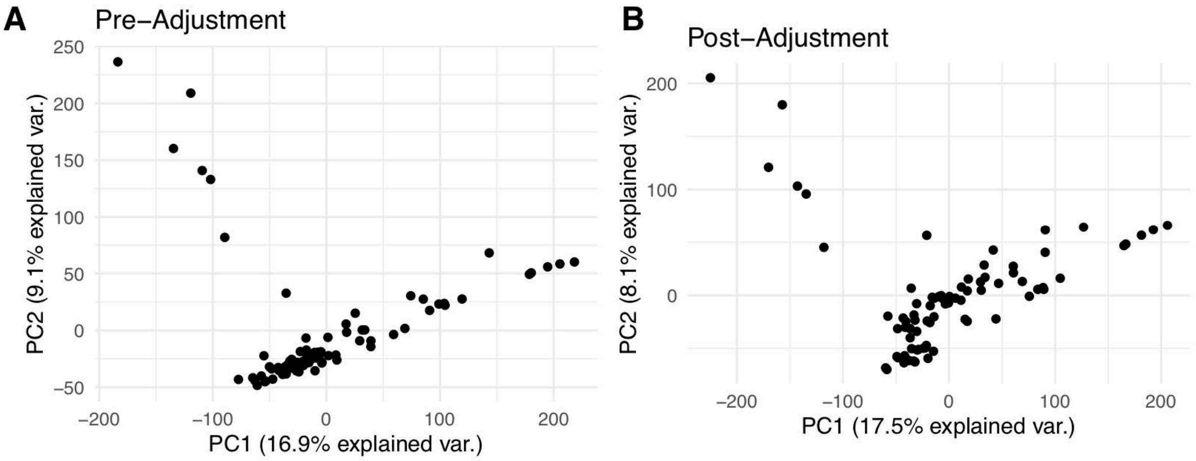

Supplementary Figure S3. Validation of findings in the GLASS cohort. Box plots of EGFR mRNA expression (normalised) with IQR boxed and median denoted by horizontal line, versus EGFR amplification status in the primary or recurrent tumour **(A)**; p value is denoted by two-sided Wilcoxon rank sum test. **(B)** Analogous box plots describing EGFR gene signature score versus EGFR amplification status in primary and recurrent tumours.

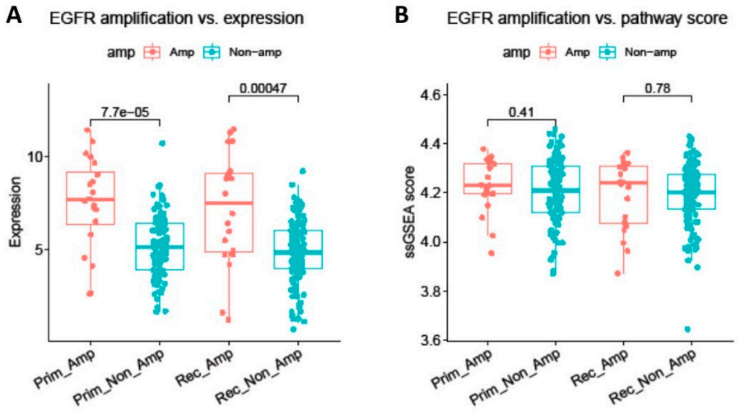

Supplementary Tables 1-4 in file supplementary_tables.xlsx: Listing of differentially expressed genes, p values, and log2 fold changes between comparisons of primary EGFR amplified tumours vs. primary EGFR non-amplified tumours **(1)**; recurrent EGFR amplified tumours vs. recurrent EGFR non-amplified tumours **(2)**; EGFR amplified primary tumours vs. EGFR amplified recurrent tumours **(3)**; and EGFR non-amplified primary tumours vs. EGFR non-amplified recurrent tumours **(4)**.
